# The Use of Natural Fiber-Rich Food Product Is Safe and Reduces Aberrant Crypt Foci in a Pre-Clinical Model

**DOI:** 10.3390/nu13082708

**Published:** 2021-08-06

**Authors:** Luane Aparecida do Amaral, Taina da Silva Fleming de Almeida, Gabriel Henrique Oliveira de Souza, Adrivanio Baranoski, Rafael Souza Maris, Felipe Francisco Bittencourt Junior, Bruna Paola Murino Rafacho, Antonio Carlos Duenhas Monreal, Cândida Aparecida Leite Kassuya, Andréia Conceição Milan Brochado Antoniolli-Silva, Elisvânia Freitas dos Santos, Rodrigo Juliano Oliveira

**Affiliations:** 1 Center for Studies in Stem Cells, Cell Therapy and Toxicological Genetics–CeTroGen, University Hospital Maria Aparecida Pedrossian, Federal University of Mato Grosso do Sul, Campo Grande 79080-190, Brazil; luapamaral@hotmail.com (L.A.d.A.); adrivaniobaranoski@yahoo.com.br (A.B.); andreia@corporesanosaude.com.br (A.C.M.B.A.-S.); 2 Postgraduate Program in Health and Development in the Midwest Region, Medical School, Federal University of Mato Grosso do Sul, Campo Grande 79070-900, Brazil; elisvania@gmail.com; 3 Postgraduate Program in Biotechnology, Faculty of Pharmaceutical Sciences, Food and Nutrition, Federal University of Mato Grosso do Sul, Campo Grande 79070-900, Brazil; tainaafleming@gmail.com (T.d.S.F.d.A.); brunapaola@gmail.com (B.P.M.R.); 4 Faculty of Pharmaceutical Sciences, Food and Nutrition, Federal University of Mato Grosso do Sul, Campo Grande 79070-900, Brazil; g.henrique99@hotmail.com; 5 Clinical Analysis Laboratory, University Center of Grande Dourados, Dourados 79824-900, Brazil; rafaelsouza_maris@hotmail.com (R.S.M.); fb_biomed@hotmail.com (F.F.B.J.); 6 Postgraduate Program in Pharmaceutical Sciences, Faculty of Pharmaceutical Sciences, Food and Nutrition, Federal University of Mato Grosso do Sul, Campo Grande 79070-900, Brazil; 7 Três Lagoas Pedagogical Center, Federal University of Mato Grosso do Sul, Três Lagoas 79600-080, Brazil; monreal.tocarlo@gmail.com; 8 Faculty of Health Sciences, University of Grande Dourados, Dourados 79825-900, Brazil; candida2005@gmail.com

**Keywords:** chemoprevention, 1,2-dimethylhydrazine, aberrant crypt foci, functional foods, dietary fibers

## Abstract

Background: Colorectal cancer is a highly prevalent disease, requiring effective strategies for prevention and treatment. The present research aimed to formulate a natural fiber-rich food product (NFRFP) and to evaluate its safety, toxicogenetics, and effects on aberrant crypt foci induced by 1,2-dimethyl-hydrazine in a preclinical model. Methods: A total of 78 male Wistar rats were distributed in six experimental groups: negative control, positive control (1,2-Dimethylhydrazine—40 mg/Kg), and four groups fed with 10% NFRFP: NFRFP, pre-treatment protocol, simultaneous treatment, and post-treatment protocol. Results: The NFRFP was shown to be a good source of fibers and did not change biometric, biochemical, hematological, and inflammatory parameters, and did not induce signs of toxicity and genotoxicity/carcinogenicity. NFRFP exhibited a chemopreventive effect, in all protocols, with damage reduction (% DR) of 75% in the comet test. NFRFP reduced the incidence of aberrant crypt outbreaks by 49.36% in the post-treatment protocol. Conclusions: The results suggest the applicability of NFRFP in the human diet due to potential production at an industrial scale and easy technological application in different products, since it could be incorporated in food without altering or causing small changes in final product sensory characteristics.

## 1. Introduction

Colon cancer exhibits high metastatic potential and it is an important public health issue as it ranks third in incidence and second in mortality among all cancers in the world, in both sexes [1]. Lifestyle and eating habits directly influence the risk and/or prevention of the development of this disease [2].

The Western diet, rich in red and processed meat, and low in fruits and vegetables [3], changes the intestinal microbiota and can trigger inflammatory processes that are associated with the development of cancer [4]. Whole cereals and grains, which are common in the Mediterranean diet, are good sources of dietary fiber associated with reduced risk of colorectal cancer [5,6,7].

Dietary fibers are non-digestible components that contribute to: (1) acceleration of the passage of metabolic waste products through the body; (2) adsorption of toxins; (3) maintaining the integrity of the digestive tract; (4) substrate for synthesis of short-chain fatty acids that provide energy for colonocytes; and (5) reduction of intestinal colonization by bacteria responsible for metabolizing and/or releasing pro-carcinogens and carcinogens [6,8,9,10]. Among the fibers possessing this capacity, those present in brown flaxseed [11], chia [12], and green banana flour [13,14], oats [15], and quinoa [16] stand out. Although textured soy protein does not have large amounts of dietary fiber, this food is resistant to digestion and absorption in the small intestine due to its protein constitution, which in turn can modulate the effects of prebiotics on the intestinal microbiota and prevent colorectal cancer [17].

Considering the high prevalence of colorectal cancer [1], new formulations based on products rich in fibers used in the prevention or as adjuvant treatments of this type of neoplasia are of paramount need. Thus, this research aimed to formulate a natural fiber-rich food product (NFRFP) and to evaluate its safety, toxicogenetics, and effects on aberrant crypt foci induced by 1,2-dimethyl-hydrazine in a pre-clinical model.

## 2. Materials and Methods

### 2.1. Chemical Agent

For the induction of aberrant crypt foci (ACF), chemical agent 1,2-Dimethylhydrazine (DMH) (Sigma^®^, St. Louis, MO, USA, CAS No. 306-37-6) was used at a dose of 40 mg/kg of body weight (BW), diluted in aqueous solution of ethylenediamine tetra-acetic acid (EDTA—0.37 mg/mL), with pH correction to 6.5 by adding 0.1 N NAOH [18], intraperitoneally (ip), with modification by Limeiras et al. [19], Navarro et al. [14], Pamplona-Silva et al. [11], and Pesarini et al. [10]. Two doses were administered per week for two weeks [20,21,22,23].

### 2.2. Preparation of Natural Fiber-Rich Food Product

The natural fiber-rich food product (NFRFP) is a mixture of brown flaxseed (Natubom^®^, Campo Grande, Brazil, Lot 2502), chia (Natubom^®^, Campo Grande, Brazil, Lot 1902), green banana flour (Natubom^®^, Campo Grande, Brazil, Lot 0701), oats (Natubom^®^, Campo Grande, Brazil, Lot 1412), quinoa (Natubom^®^, Campo Grande, Brazil, Lot 0101) and textured soy protein (Natubom^®^, Campo Grande, Brazil, Lot 702), acquired in commercial establishments in the city of Campo Grande, MS, Brazil. All ingredients were ground and mixed in the same proportion. Next, NFRFP was added to crushed commercial chow (Nuvilab^®^, Colombo, Brazil) in the proportion of 10%. After homogenization, the modified chow was moistened by adding filtered water, pelleted, and dried in an oven with air circulation at 35 °C for 12–14 h.

### 2.3. Animals and Environmental Conditions

Seventy-eight male Wistar rats of reproductive age, i.e., approximately 10–12 weeks old, from the Central Vivarium of the Biosciences Institute of the Federal University of Mato Grosso do Sul, Campo Grande, MS, Brazil, were distributed in six experimental groups (*n* = 13 animals/group) after an adaptation period of 7 days.

Animals were kept in pairs or trios in polypropylene boxes covered with autoclaved shavings. Luminosity and room temperature were controlled (12 h of light: 12 h of dark) around 22 °C ± 2 and 55% ± 5 of humidity. Animal feeding consisted of filtered water and commercial chow (Nuvilab^®^, Colombo, Brazil) or commercial chow (Nuvilab^®^, Colombo, Brazil) plus 10% NFRFP, ad libitum.

The experiment was carried out in accordance with the Ethical Principles in Animal Research and approved by the Ethics Committee on the Use of Animals at the Federal University of Mato Grosso do Sul under opinion nº 1000/2018.

### 2.4. Experimental Design

**Figure 1:**
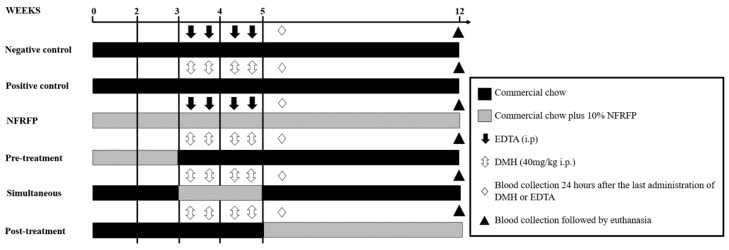
Experimental design. NFRFP—Natural Food Product Rich in Fiber; EDTA—ethylenedia-mine tetra-acetic acid; DMH—1,2-dimethylhydrazine; i.p.—intraperitoneal.

Animals in the Negative Control group received commercial chow for twelve weeks and in the 3rd and 4th weeks received two doses of aqueous EDTA solution (0.37 mg/mL) in the proportion of 1 mL/100 g of (BW; i.p.). Animals in the Positive Control group were treated in the same way as the negative control, replacing EDTA with 40 mg/kg 1,2-dimethylhydrazine (DMH) solution (BW; i.p.). Animals in the NFRFP group were treated as the negative control but were fed with 10% NFRFP chow. Animals in the Pretreatment group received commercial chow plus 10% NFRFP for two weeks (1st and 2nd weeks). Then, they started to receive commercial chow (3rd to 12th week). In the 3rd and 4th weeks, they received two doses/week of DMH (40 mg/Kg, BW, i.p.). Animals in the Simultaneous group received commercial chow in the 1st and 2nd weeks, then in the 5th to 12th weeks. In the 3rd and 4th weeks, the animals received two doses/week of DMH (40 mg/Kg, BW, i.p.) and were fed with a 10% NFRFP chow. Animals in the Post-treatment group received commercial chow from the 1st to the 4th week and then started to receive chow plus 10% NFRFP from the 5th to the 12th week. At the 3rd and 4th weeks, the animals received two doses/week of DMH (40 mg/Kg, BW, i.p.) (Figure 1).

The present experimental design is common in the field area [14,19,21,24,25,26] as it might indicate the mechanism of action of dietary fiber in the prevention and/or repair of DNA damage and other biomarkers for cancer. Thus, pre-treatment and simultaneous treatment protocols suggest a preferentially demutagenic effect and the post-treatment protocol a bioantimutagenesis effect [27,28,29,30].

Individual body weight and food consumption were quantified twice a week during the experimental period. Twenty-four hours after the last administration of EDTA or DMH, a sample of 20 µL of peripheral blood was collected, by puncture of the caudal vein, to perform the comet assay and 20 µL to perform the micronucleus assay (Figure 1).

At the end of the 12th week, animals were subjected to inhaled anesthesia with isoflurane to collect blood by retro-orbital puncture. Then, the animals were euthanized by overdose of the same anesthetic. Subsequently, animals were submitted to thoracotomy and laparotomy for the collection of organs and, in particular, the intestine for evaluation of the ACF (Figure 1).

### 2.5. Chemical Analysis of Feed and Natural Fiber-Rich Food Product

Humidity was determined in an oven at 105 °C until constant weight (AOAC, 2011). Ashes were determined in a muffle furnace (550 °C) [31]. Total lipids were determined by the method of Bligh & Dyer [32]. Proteins were evaluated using the total nitrogen content of the sample, assessed using the Kjeldahl method and determined at the semi-micro level [31]. Nitrogen to protein conversion factor of 6.25 was applied. Total dietary fiber content was assessed using the AOAC 985.29 method [31].

Carbohydrate content was assessed by theoretical calculation (by difference) using the formula % Carbohydrates = 100 − (% moisture + % protein + % lipids + % ash + % dietary fiber). The total caloric value (kcal) was calculated using the following values: lipids (9.03 kcal/g), protein (4.27 kcal/g), and carbohydrates (3.82 kcal/g) [33]. All analyses were performed in triplicate.

### 2.6. Biological Tests

#### 2.6.1. Evaluation of Genotoxicity and Antigenotoxicity

##### Comet Assay

This assay was conducted according to Singh et al. (1988) with modifications by Navarro et al. [34]. Twenty microliters of peripheral blood were homogenized with 120 μL of low melting point agarose (Low melting point—LMP—1.5%) at 37 °C. Then, this mixture was deposited on a slide pre-covered with normal agarose (5%) and covered by a glass coverslip. Slides were cooled to 4 °C for 20 min and the coverslips were removed. Slides were immersed in a lysis solution (89.0 mL of lysis stock (2.5 M NaCl, 100.0 mM EDTA, 10.0 mM Tris, pH 10.0 corrected with solid NaOH, 890.0 mL of distilled water and 1% sodium lauryl sarcosinate), 1.0 mL of Triton X-100 and 10.0 mL of DMSO) for 1 h. Afterwards, slides were transferred to the electrophoresis buffer for denaturation (300.0 mM NaOH and 1.0 mM EDTA, prepared from a stock solution of 10.0 N NaOH and EDTA 200.0 mM pH10.0) for 20 min and, subsequently, electrophoresis was performed with buffer pH > 13 at 4 °C for 20 min. After electrophoresis, slides were neutralized with 15.0 mL of neutralization solution (0.4 M Tris and 950 mL deionized water—pH 7.5) for 15 min and dried in open air and fixed in absolute ethyl alcohol for 10 min. Slides were stained with 100 µL of ethidium bromide. The nucleoids were photographed under a fluorescence microscope (Leica^®^, DMi8, Wetzlar, Germany) with a magnification of 200×. Subsequently, 200 nucleoids/animal were analyzed in the CometScore 2.0.0.38 TriTek program. The parameters used were the percentage of DNA in the tail and the moment of the tail.

An internal test control was performed with B16F10 cells treated with doxorubicin (5 µM) for 3 h. Cell cultivation was performed according to Navarro et al. [35]. The slides were submitted to the same electrophoresis run as the peripheral blood slides.

##### Peripheral Blood Micronucleus Assay

The peripheral blood micronucleus assay was carried out as described by Hayashi et al. [36] and modified by Navarro et al. [34]. For this purpose, a slide covered with 20 μL of Acridine Orange (1.0 mg/mL) received 20 μL of peripheral blood from the caudal vein. The slide was covered by a coverslip and stored in a freezer (−20 °C) for a minimum period of seven days. The analysis was performed in an epifluorescence microscope (Motic^®^, BA410 FL, Vancouver, BC, Canada), at 40× magnification, with excitation filter 420–490 nm and barrier filter 520 nm. A total of 2000 cells/animal were analyzed.

#### 2.6.2. Testing of Aberrant Crypt Foci

After euthanasia, large intestines were collected. These were opened by the mesenteric line and fixed in styrofoam. Subsequently, the intestines were fixed in a 10% formalin buffered solution for a minimum period of 24 h. At the time of analysis, each segment of the colon was stained with 10% methylene blue solution for 10 min and deposited on a slide with the mucosa facing upwards. The analysis was performed using a 10× magnification bright field optical microscope. The entire mucosa was evaluated for the identification and quantification of ACF. The identification of ACF was based on the criteria used by Bird [37]: (1) focus consisting of a single crypt—the aberrant crypt is covered by a thick epithelial layer, with an elliptical lumial opening and of a larger size (at least 2×) than those of the surrounding normal crypts; (2) focus with two or more crypts—the aberrant crypts form separate blocks and occupy an area larger than that occupied by an equivalent number of crypts of normal morphology. There is no presence of normal crypts separating aberrant crypts within these foci. The ACF were analyzed according to the occurrence of 1–3 crypts/outbreak, 4–8 crypts/outbreak, and more than 9 crypts/outbreak. For statistical analysis, the total number of ACF, of aberrant crypts by foci, and the crypt/focus ratio were considered [38].

#### 2.6.3. Calculation of Damage Reduction Percentage (% DR)

The percentage of NFRFP damage reduction in DMH-induced injuries was calculated according to Manoharan and Banerjee [39], with modifications by Pesarini et al. [10], as follows:

$$\%\mathrm{DR}=\frac{\mathrm{Average}\;\mathrm{of}\;\mathrm{the}\;\mathrm{positive}\;\mathrm{control}\;-\;\mathrm{Average}\;\mathrm{of}\;\mathrm{the}\;\mathrm{associated}\;\mathrm{group}}{\mathrm{Average}\;\mathrm{of}\;\mathrm{the}\;\mathrm{positive}\;\mathrm{control}\;-\;\mathrm{Average}\;\mathrm{of}\;\mathrm{the}\;\mathrm{negative}\;\mathrm{control}}\times 100$$

#### 2.6.4. Hematological and Biochemical Parameters

Hematological analyzes were carried out in a KX-21 automation unit (Sysmex^®^), according to the manufacturer’s recommendations, and revised on a slide by means of differential counting (leukocytes) [40] and observation of cell morphology/staining [40].

Peripheral blood collected by retro-orbital puncture was placed in a tube with separating gel (BD^®^, Curitiba, Brazil, CAT: 367986, lot: 6305645-2017-10-31). Then, it was centrifuged at 2000 rpm for 10 min (Fanem^®^, Excelsa 3, São Paulo, Brazil). Serum was separated for biochemical analyses performed on Cobas C111 using commercial kits (Roche^®^, Mannheim, Germany, Batch: aspartate aminotransferase—AST 41777901, alanine aminotransferase—ALT 42120501, total protein 3824560, albumin 38244901, serum urea 42549401, creatinine 38184001 uric 34263401, glucose 40900901, α-amylase 42941601, cholesterol 41613201, HDL cholesterol 39303201 and triglycerides 40929901) according to the manufacturer’s recommendations.

The parameters evaluated included total cholesterol, HDL cholesterol, glucose, α—amylase, lipase, markers of liver function (ALT and AST), markers of renal function (urea, creatinine, and uric acid), and protein profile (albumin and total proteins).

#### 2.6.5. Quantification of IFN-γ, IL-6, IL-10, IL-12p70, MCP-1 and TNF-α Cytokine Expression

Peripheral blood collected by retro-orbital puncture was placed in a tube with separating gel (BD^®^, CAT: 367986, lot: 6305645-2017-10-31). Then, it was centrifuged at 2000 rpm for 10 min (Fanem^®^, Excelsa 3, São Paulo, Brazil). The serum was separated for cytokine quantification, using the Cytometric Beads Array Kit—CBA (CAT: 552364, Lot: 8171797; BD^®^), following the manufacturer’s instructions, using a Cytoflex Beckman Coulter^®^ flow cytometer. For this purpose, 50 µL of serum from each animal was incubated with 50 µL of marker (Mouse Inflammation PE Detection Reagent) together with the marked capture spheres (Capture Beads) for 2 h. Subsequently, the mixture was centrifuged at 1500 rpm for 5 min. The supernatant was discarded. The precipitate was homogenized with 1 mL of wash buffer (Wash buffer) and a new centrifugation and supernatant disposal were performed. At the end, the precipitate was resuspended in 300 µL of wash buffer and 10,000 spheres were evaluated in a Cytoflex Beckman Coulter flow cytometer to determine the amount of each cytokine. The data were calculated using simple linear regression calculation on a standard curve to obtain the values in pg/mL.

### 2.7. Statistical Analysis

Chemical analysis results were expressed as mean ± standard deviation and other variables as mean ± standard error. Parametric distribution data were analyzed by ANOVA/Tukey and non-parametric Kruskal–Wallis/Dunn. The level of significance adopted was *p* < 0.05.

## 3. Results

### 3.1. Chemical Analysis of Diets and Evaluation of Food Intake

Chemical analysis of commercial chow and commercial chow plus 10% NFRFP showed that the addition of NFRFP reduces (*p* < 0.05) the moisture and amount of carbohydrates by 19.45% and 7.7%. The amount of dietary fiber increased by 20.94%. Ashes, protein, lipids, and calories did not change significantly (*p* > 0.05) (Table 1).

Chemical analysis of NFRFP showed that it contains 7.60 ± 0.16 g/100 g of moisture, 3.44 ± 0.06 g/100 g of ashes, 17.65 ± 0.30 g/100 g of protein, 11, 81 ± 0.13 g/100 g of lipids, 40.76 ± 0.76 g/100 g of carbohydrates, 337.72 ± 1.27 g/100 g of calories and 18.75 ± 0.62 g/100 g of total fiber (Table 1).

Average food intake did not differ among the experimental groups (*p* > 0.05). The lowest food intake was observed in the simultaneous treatment group (19.83 ± 2.61) and the highest was observed in the negative control (24.53 ± 0.91) (Supplementary Figure S1).

### 3.2. Effects of Feed Plus 10% NFRFP and Feeding Protocols on Biometric Parameters

Animals started the experiment period with similar body weights (*p* > 0.05). Final body weight and weight gain did not show statistically significant differences among the experimental groups regardless of diet and treatment protocol (*p* > 0.05). Absolute and relative weights of heart, lung, liver, spleen, kidneys, right epididymis, and right testicle did not present significant differences when compared to the negative control group (*p* > 0.05) (Supplementary Table S1).

### 3.3. Genotoxicity Tests

**Figure 2:**
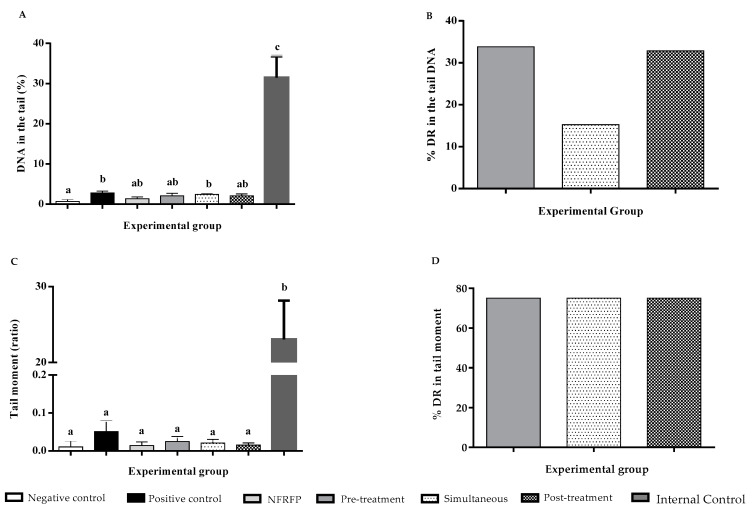
Chemopreventive action of Natural Fiber-Rich Food Product (NFRFP), by the comet assay, in different protocols for inducing DNA damage by 1,2-Dimethylhydrazine. (**A**) % DNA in the tail; (**B**) % Reduction of DNA damage in the tail DNA; (**C**) Moment of the tail; (**D**) % Reduction of damage in tail moment. Internal Control—was performed with B16F10 cells treated with doxorubicin (5 µM). Different letters indicate statistically significant differences (Statistical Test: ANOVA/Tukey; *p* < 0.05).

The Comet test showed that DMH is capable of increasing the frequency of genomic lesions. Thus, there was an increase of 4.09× in % tail DNA and 5× in moment of the tail (*p* < 0.05) (Figure 2A,C).

NFRFP did not cause changes (*p* > 0.05) in the frequency of % tail DNA or in the moment of the tail when compared to the negative control (*p* > 0.05) (Figure 2C).

The association protocols demonstrated that NFRFP exhibits a chemopreventive effect (*p* < 0.05). The % DR in % tail DNA was 33.81%, 15.24%, and 32.86% for pre-treatment, simultaneous treatment, and post-treatment, respectively (Figure 2B). Regarding % DR for the tail moment, results showed 75% for all protocols analyzed (*p* < 0.05) (Figure 2D).

Figure 2A,C show (in the last bar of the histogram) values of% DNA in the tail and Tail Moment for an internal control of the test, performed with B16F10 cells treated with Doxorubicin (5 µM), which presented the values of 31.44 ± 5.33 and 23.03 ± 5.12, respectively.

**Figure 3:**
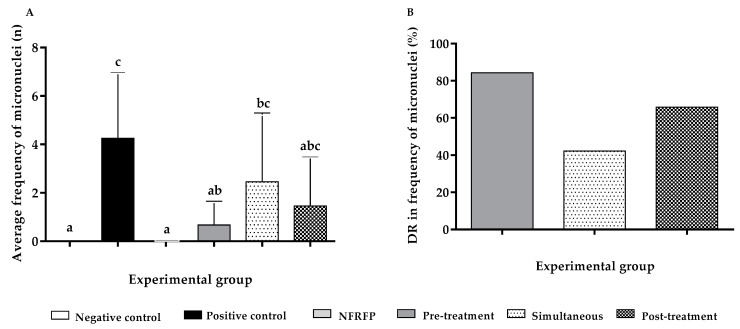
Chemopreventive action of the Natural Fiber-Rich Food Product (NFRFP), by the micronucleus assay, in different protocols for inducing DNA damage by 1,2-Dimethylhydrazine. (**A**) Average frequency of micronuclei; (**B**) DNA Damage Reduction Percentage. Different letters indicate statistically significant differences (Statistical Test: Kruskal-Wallis/Dunn; *p* < 0.05).

The frequency of micronuclei in the positive control increased (*p* < 0.05) by 4.25 times in relation to the negative control. The NFRFP did not induce an increase in the frequency of micronuclei (*p* > 0.05) (Figure 3A). The chemopreventive effect was observed only for the pre-treatment protocol (*p* < 0.05) (Figure 3A) with % DR of 84.31% (Figure 3B). The simultaneous and post-treatment protocols did not show chemopreventive activity (*p* > 0.05) (Figure 3A) and the % DR were 42.24% and 65.76%, respectively (Figure 3B).

### 3.4. Aberrant Crypt Foci

DMH increased (*p* < 0.05) the frequency of foci by 72.80× (*p* < 0.05) (Table 2). NFRFP did not increase the frequency of AFC when compared to the negative control group (*p* > 0.05) (Table 2).

The chemoprotective effect of NFRFP was observed, significantly, for pre- and post-treatment in the occurrence of 1–3 crypts/foci (*p* < 0.05). Regarding 4–8 crypts/foci, all protocols tested were chemopreventive (*p* < 0.05). For the occurrence of more than nine crypts, none of the protocols demonstrated chemoprevention (*p* > 0.05) (Table 2). A general analysis allows us to infer that only the post-treatment protocol was efficient in reducing the AFC induced by DMH and % DR was 49.36%. However, pre-treatment and simultaneous treatment protocols did not show a significant effect, with % DR of 25.18% and 32.31%, respectively.

### 3.5. Evaluation of Biochemical and Hematological Parameters

Hematological analysis showed that platelet concentration was lower than reference values for Wistar rats (CHARLES RIVER LABORATORIES, 2008; DANTAS et al., 2006) in the negative control group, NFRFP and pretreatment groups. RDW was lower than the reference value for the pre-treatment group. Statistical analysis demonstrated that there were no significant differences between the experimental groups and the negative control regarding the number of leukocytes, neutrophils, and eosinophils, hemoglobin concentration, and RDW (*p* > 0.05). The simultaneous treatment group showed an increase (*p* < 0.05) in the concentration of erythrocytes. Simultaneous and post-treatment groups showed an increase (*p* < 0.05) for hematocrit, platelets, and monocytes. The post-treatment group showed a reduction (*p* < 0.05) in the frequency of lymphocytes in relation to the negative control (Table 3).

Biochemical tests demonstrated that albumin level was higher than reference values for Wistar rats (CHARLES RIVER LABORATORIES, 2008; DANTAS et al., 2006) in NFRFP and pretreatment groups. Cholesterol was lower than the reference value for the NFRFP group. HDL cholesterol was higher than the reference values in the negative control, pretreatment, and simultaneous treatment groups. Triglyceride concentrations were lower than the reference values for the NFRFP groups, pre-treatment, and simultaneous treatment. Statistical analysis showed that there were no differences among the experimental groups and the negative control group, except for the significant reduction in AST observed in the pre-treatment and post-treatment groups and for the reduction of cholesterol for the NFRFP group (*p* < 0.05) (Table 4).

### 3.6. Quantification of IFN-y, IL-6, IL-10, IL-12p70, MCP-1 and TNF-α Cytokine Expression

**Figure 4:**
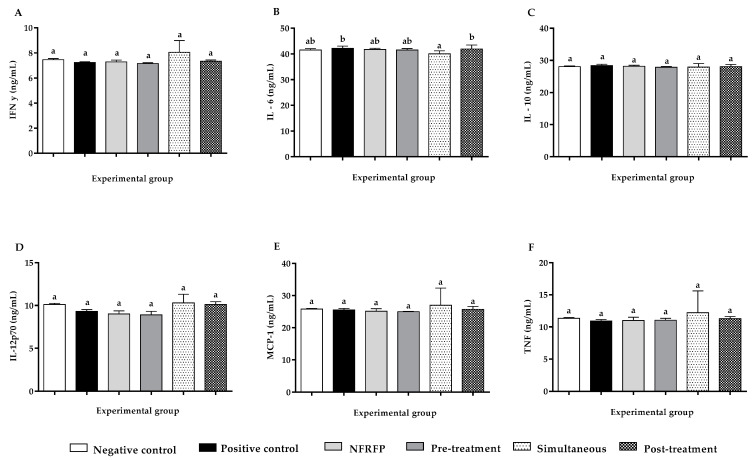
Effect of consumption of the Natural Fiber Rich Food Product (NFRFP) on the expression of the cytokines IFN-y (**A**), IL-6 (**B**), IL-10 (**C**), IL-12p70 (**D**), MCP-1 (**E**) and TNF-α (**F**) in rats treated with 1,2-dimethylhydrazine (DMH). Different letters indicate statistically significant differences (Statistical Test: ANOVA/Tukey; *p* < 0.05).

The evaluation of the systemic inflammatory process demonstrated that there were no variations in the concentration of IFN-y, IL-6, IL-10, IL-12p70, MCP-1, and TNF-α among different experimental groups, except for IL-6 levels among positive control and post-treatment groups compared to the simultaneous group. However, none of these groups differ from the negative control (Figure 4).

## 4. Discussion

According to the Brazilian National Health Surveillance Agency [41], high fiber content products are those containing ≥6 g/100 g. NFRFP presented 18.55 g/100 g. Therefore, it is considered a good source of fiber, representing a natural food product to be included in the human diet. The average recommended daily fiber intake for adults, of both sexes, aged 19–50 years, is 31.5 g/day [42]. Therefore, the consumption of a 50 g portion of NFRFP supplies 59.52% of the daily dietary fiber requirements.

Regarding humidity, commercial chow and chow plus 10% NFRFP were statistically different (*p* < 0.05). Such a difference may have occurred due to the drying process of NFRFP chow. Elias et al. [43] reported that the moisture content is related to drying methods, binomial time/temperature, and storage period.

ANVISA [44] determines that flours might contain a maximum of 15 g/100 g of moisture, aiming to prevent the proliferation of microorganisms and increase the durability of the product. Thus, NFRFP chow displayed 7.60 g/100 g of moisture and it can be considered a flour. It was observed that the addition of NFRFP did not change the content of ashes (mineral residue), lipids, and proteins between the analyzed diets.

Regarding protein content, a food might be considered as a protein source if it presents at least 6 g of protein/100 g of prepared product or portion. Food can be considered as high in protein as long as it contains at least 12 g of protein per 100 g of prepared product or portion [41]. Thus, NFRFP might be labeled as a high protein product and might be used a complementary ingredient in food products in order to increase protein value.

The addition of NFRFP in the diet reduced the amount of chow carbohydrates by 7.7%. This characteristic is desirable since evidence showed that diets rich in carbohydrates are responsible for the development of chronic diseases, such as obesity [45,46] and cancer [47,48]. Furthermore, NFRFP increased chow total dietary fiber by 20.94%, a desired characteristic since dietary fibers may be associated with decreased incidence of colorectal cancer. Diet fiber exerts beneficial effects such as increasing fecal volume, regulating intestinal flora, and promoting the dilution and/or adsorption of possible carcinogens [6,8,9,11].

Another interesting fact is that the addition of NFRFP to the chow did not change food intake of experimental groups. Thus, it is suggested that the product is palatable, with easy technological application in different products, as it can be incorporated without changing sensory characteristics of the final product. The absence of weight change, including absolute and relative weight of organs, also allows us to infer that NFRFP did not show signs of toxicity. No changes in hematological and biochemical parameters also suggest an absence of toxicity.

Regarding biochemical evaluation, platelet counts observed were below reference values (Lima et al., 2014) for the negative control, NFRFP, and pre-treatment groups. The RDW also presented values below the reference values [49] in the pre-treatment group, as well as the plasma triglyceride concentrations. Albumin concentration was above the reference values [50] in the NFRFP and pre-treatment groups. The same was observed for HDL cholesterol in the pretreatment and simultaneous treatment groups. Taken together, it was not possible to establish any relationship between the intake of NFRFP and concentration of platelets and HDL cholesterol since variations were also observed in the control group that did not ingest NFRFP. Thus, we infer that these findings represent normal variation in rats used in the present study.

Significant differences were observed among the control and treatment groups, such as an increase in the amount of erythrocytes in the simultaneous group; increased hematocrit in the simultaneous and post-treatment groups; increase in platelets in the simultaneous and post-treatment groups; increase of monocytes in the simultaneous and post-treatment groups; reduction of lymphocytes in the post-treatment; and reduction of AST and ALT in the pre-treatment and post-treatment groups. Those changes can be considered within the reference values, not related with NFRFP ingestion nor biologically relevant. However, it is important to notice that plasma cholesterol concentration was lower in the NFRFP group compared to the control group and below the reference value for rats [51]. Thus, we suggest that NFRFP may have a hypocholesterolemic effect if consumed for 12 consecutive weeks. This conclusion is corroborated by findings in the post-treatment group, who ingested NFRFP for seven weeks and presented a tendency towards reducing plasma cholesterol concentration, and in pre-treatment and simultaneous treatment groups that presented no changes in cholesterol concentration after ingestion of NFRFP for two weeks.

Dietary fibers can reduce cholesterol by increasing intestinal transit, which prevents part of the absorption of fats [52], by assisting the capture of lipids that are more easily integrated into the fecal bolus [52,53], and by producing important metabolites such as short-chain fatty acids (acetate, propionate and butyrate) [54,55] after fermentation by specific groups of bacteria, such as lactobacilli and bifidobacteria [56]. The main short-chain fatty acid is butyrate, which is responsible for colonocyte nutrition and exhibits anticarcinogenic and anti-inflammatory potential. Butyrate acts on the intestinal epithelial barrier and modulates oxidative stress [54,57,58]. In addition, according to Anderson et al. [59], short-chain fatty acids, mainly propionic acid, decrease cholesterol synthesis in the liver, leading to cholesterol reduction in the bloodstream. Thus, we infer that the presented mechanism is implied in the hypocholesterolemic action of NFRFP for 12 weeks.

Despite the statistical analysis showing that the positive controls caused DNA damage in the comet assay (% tail DNA) and micronucleus tests, the values found have no biological relevance. This can be explained by the fact that DHM is a compound with low initiation and associated with strong promotion of colorectal carcinogenesis [60,61]. Another fact that can explain this finding is that the spleen of rats is very proficient in sequestering cells with DNA damage (genomic damage—comet; chromosomal damage—micronuclei) [62] compared to mice, for example. These data are corroborated by studies carried out with mice that demonstrated that DMH was capable of causing genotoxic damage [10,11,14,19,24]. The study by Pesarini et al. [10] demonstrated that DMH (30 mg/Kg) caused an average frequency of 99.60 ± 0.22 injured cells for every 100 cells analyzed. The micronucleus frequency in this same study was 28.20 ± 0.75 in every 2000 cells analyzed. Thus, we infer that the detection of DNA damage can vary according to the experimental model used to induce aberrant crypts as well as the dose of DMH used. We suggest that genotoxic damage can be better evaluated in studies with mice. However, the rat model is the best for evaluating aberrant crypt foci.

These results were already expected by our research group. Thus, to avoid doubts as to whether the techniques were well standardized, we simultaneously treated a negative control group and a positive control group with DHM, in the Swiss mouse model (data not shown) and ran the slides together with the slides of the present study. In Swiss mice, the frequency of genetic damage in the positive control was at least 2× more than the frequency of the negative control, a condition necessary for a compound to be considered positive [24,63,64]. This fact may occur because, according to Rabello-gay et al. [65], the spleen of rats and humans are more efficient at sequestering cells with DNA damage compared to the capacity of mice. Therefore, this may be the explanation for the lesions being observed in mouse models and not in rats.

Despite the low capacity of DMH to induce detectable DNA damage measured by comet and micronucleus techniques, this compound was an efficient promoter of intestinal chemical carcinogenesis since it increased the frequency of aberrant crypt foci in Wistar rats by 72.8×. The frequency of foci in the negative control group and NFRFP did not differ, which reinforces the safety of using this compound in the diet. In addition, NFRFP was able to significantly reduce the frequency of aberrant crypt foci in the post-treatment group (% DR = 49.36). Furthermore, a tendency was found in reducing lesions in the pre-treatment and simultaneous treatment groups with % DR rates of 25.18 and 32.31. Despite the absence of significant differences, these values are interesting in the cancer prevention context and can be reevaluated in other studies, including other experimental models.

The pre-treatment and simultaneous treatment protocols are indicative of which substances can act by desmutagenesis, meaning a compound or products of its metabolism that can act directly on the damage inducer by chelation or enzymatic inactivation [10,11,19,24,27,28,29,66,67]. This mechanism is described for different foods or food components [10,11,14,19,21,24,25,26,28] and fibers [10,11,14,19,20,24,26,29]. The post-treatment protocol, on the other hand, allows the evaluation of compounds with bioantimutagenic actions that are capable of modulating repair enzymes favoring the correction of DNA damage [27,28,29,30,68].

NFRFP acts by bioantimutagenesis since it prevented the occurrence of aberrant crypts foci in the post-treatment protocol. In this case, the crypts were induced for two consecutive weeks, with applications of DMH, and, at the end of the induction, animals started receiving NFRFP. This result suggests that this compound or its metabolites are capable of modulating cellular repair machinery in order to reverse DNA damage and, even partially, the development of aberrant colorectal cancer foci crypts. Thus, we demonstrate the pioneering and original nature of the NFRFP.

Fiber-rich compounds, such as NFRFP, can exhibit chemopreventive effect. The chemopreventive properties of dietary compounds involve multiple molecular and biochemical mechanisms that lead to the inhibition of cell growth, tumor initiation, adhesion, migration, angiogenesis, apoptosis, interaction with the intestinal microbiota, regulation of cell signal transduction pathways and xenobiotic metabolizing enzymes [69]. Thus, it is inferred that NFRFP can act in this line by promoting the prevention, suppression, or reversal of carcinogenesis in its early stages.

It is known that ACF are induced by an inflammatory response. In summary, DMH is hydrolyzed in the liver to produce methylazoxymethane, which conjugates with β-glucuronic acid and is carried to the intestinal lumen. In the intestinal lumen, bacterial β-glucuronidase releases the active metabolite of DMH, azoxymethane [70]. This metabolite triggers a mild inflammatory reaction that increases cell proliferation in the colon mucosa. When administered chronically, this drug leads to continuous proliferation and the induction of GC → AT transitions in genes, such as β-catenin and Kras, triggering the induction of colorectal carcinogenesis [71].

Based on the previous concept, the profile of some cytokines that are involved in the inflammatory process and its resolution, cell proliferation, and differentiation were evaluated. However, our results showed that, according to the present protocol, cancer induction with DMH and/or NFRFP consumption does not modify serum concentrations of IFNγ, IL-6, IL-10, IL-12p70, MCP-1, and TNF-α. The consulted literature presented few data that correlated the cytokine dosage in this model of induction of aberrant crypt foci by DMF. However, Li et al. [72] demonstrated that germinated brown rice is capable of reducing IL-6 (<0.25 ng/mL) and TNF-α (<0.35 ng/mL), in contrast with the findings of the present study. Thus, further research might clarify these observations.

Taking into account the benefits of NFRFP, we believe that it might be of industrial interest by adding fiber sources to foods and beverages without modifying or causing major modifications to the organoleptic characteristics, with potential use for healthy individuals and for those affected by colorectal cancer. In addition, NFRFP presents a low cost and is derived from foods already part of the human diet.

## 5. Conclusions

NFRFP does not cause toxicity and/or genotoxicity/carcinogenicity, nor changes in the biochemical, hematological, and inflammatory profiles (isolated or in association with DMH), except for the reduction of cholesterol when consumed for at least 12 consecutive weeks. This formulation presented a chemopreventive effect and reduced the progression of aberrant crypt foci in the post-treatment protocol. These results open avenues to studying the applicability of NFRFP in the human diet due to the possibility of production on an industrial scale and easy technological application in different products. NFRFP can be incorporated into food without altering sensory characteristics of the final product.

## Figures and Tables

**Table 1:** Chemical analysis of commercial chow, chow plus 10% NFRFP and NFRFP.

Parameters	Commercial Chow Nuvilab^®^	Commercial Chow Plus 10% NFRFP	NFRFP
	Mean ± SD	Mean ± SD	Mean ± SD
Humidity (g/100 g)	9.47 ± 0.16 ^b^	7.14 ± 0.17 ^a^	7.60 ± 0.16
Ashes (g/100 g)	7.00 ± 0.07 ^a^	6.96 ± 0.18 ^a^	3.44 ± 0.06
Protein (g/100 g)	20.14 ± 0.08 ^a^	21.19 ± 0.49 ^a^	17.65 ± 0.30
Lipids (g/100 g)	3.80 ± 0.04 ^a^	4.74 ± 0.47 ^a^	11.81 ± 0.13
Carbohydrates (g/100 g) *	42.20 ± 0.07 ^b^	38.95 ± 0.68 ^a^	40.76 ± 0.76
Calories (kcal/100 g)	281.57 ± 0.47 ^a^	282.06 ± 0.55 ^a^	337.72 ± 1.27
Total food fiber (g/100 g)	17.38 ± 0.04 ^a^	21.02 ± 0.88 ^b^	18.75 ± 0.62

NFRFP—Natural Fiber-Rich Food Product; SD: Standard Deviation. Data expressed on wet basis. Different letters in the same line indicate statistically significant differences (Statistical Test: Student’s *t*; *p* < 0.05). * Calculated by difference.

**Table 2:** Effect of consumption of Natural Fiber-Rich Food Product (NFRFP) on the frequency of aberrant crypts foci and reduction of damage to the colon of rats treated with 1,2-dimethylhydrazine (DMH).

Group/Treatment	ACF Number			Total ACF Number	%DR
	1–3 Crypts	4–9 Crypts	≥9 Crypts		
Negative Control	1.00 ± 0.46 ^a^	0.25 ± 0.18 ^a^	0.00 ± 0.00 ^a^	1.25 ± 0.52 ^a^	–
Positive Control	50.27 ± 10.42 ^c^	36.82 ± 5.65 ^b^	3.91 ± 1.15 ^b^	91.00 ± 15.82 ^c^	–
NFRFP	3.08 ± 1.04 ^a^	1.69 ± 0.58 ^a^	0.15 ± 0.15 ^a^	4.92 ± 1.62 ^a^	–
Pre-treatment	28.20 ± 4.61 ^b^	37.30 ± 7.30 ^b^	2.90 ± 1.32 ^ab^	58.40 ± 11.45 ^bc^	25.18
Simultaneous	33.44 ± 4.49 ^bc^	27.33 ± 5.11 ^b^	1.22 ± 0.43 ^ab^	62.00 ± 9.07 ^bc^	32.31
Post-treatment	24.20 ± 3.67 ^b^	21.50 ± 3.18 ^b^	1.00 ± 0.39 ^ab^	46.70 ± 5.70 ^b^	49.36

NFRFP—Natural Fiber-Rich Food Product; % DR—Percentage of Damage Reduction. Different letters indicate statistically significant differences (Statistical Test: ANOVA/Tukey; *p* < 0.05).

**Table 3:** Effect of consumption of Natural Fiber-Rich Food Product (NFRFP) on hematological parameters in rats treated with 1,2-dimethylhydrazine (DMH).

Parameters	Experimental Groups						Reference Value
	Negative Control	Positive Control	NFRFP	Pre-Treatment	Simultaneous	Pos-Treatment	
Leucocytes (10^3^/µL)	8.34 ± 0.78 ^a^	10.94 ± 1.11 ^a^	9.11 ± 0.45 ^a^	9.82 ± 0.90 ^a^	9.87 ± 0.74 ^a^	10.32 ± 1.00 ^a^	3.41–13.7 ^1^
Erythrocytes (10^6^/µL)	5.91 ± 0.51 ^a^	6.20 ± 0.27 ^a^	6.59 ± 0.08 ^ab^	6.43 ± 0.20 ^ab^	7.74 ± 0.30 ^b^	7.20 ± 0.40 ^ab^	5.4–8.5 ^2^
Hemoglobin (g/dL)	11.59 ± 0.82 ^a^	11.94 ± 0.58 ^a^	12.75 ± 0.09 ^a^	12.37 ± 0.31 ^a^	13.57 ± 0.97 ^a^	12.35 ± 1.16 ^a^	10.2–17.8 ^1^
Hematocrit (%)	34.37 ± 2.48 ^a^	35.84 ± 1.12 ^ab^	38.05 ± 0.34 ^abc^	36.66 ± 0.94 ^abc^	43.00 ± 1.37 ^c^	40.58 ± 1.52 ^bc^	23.8–51.9 ^1^
Platelets (10^3^µL)	635.40 ± 59.47 ^a^	786.80 ± 38.78 ^ab^	712.00 ± 16.33 ^a^	684.20 ± 42.43 ^a^	981.60 ± 58.60 ^b^	938.90 ± 89.64 ^b^	727–1351 ^1^
Neutrophils (%)	12.92 ± 1.55 ^ab^	16.18 ± 2.43 ^ab^	9.77 ± 0.44 ^a^	16.30 ± 1.67 ^ab^	13.11 ± 2.15 ^ab^	19.10 ± 3.24 ^b^	NF
Lymphocytes (%)	83.45 ± 1.43 ^b^	79.73 ± 2.59 ^ab^	86.35 ± 0.47 ^b^	79.33 ± 1.79 ^ab^	81.99 ± 2.27 ^ab^	75.12 ± 2.71 ^a^	43.1–93.7 ^1^
Monocytes (%)	2.08 ± 0.29 ^a^	2.64 ± 0.31 ^ab^	2.39 ± 0.14 ^ab^	3.00 ± 0.21 ^ab^	3.33 ± 0.24 ^b^	3.40 ± 0.31 ^b^	1–15.2 ^1^
Eosinophils (%)	1.08 ± 0.15 ^a^	1.46 ± 0.28 ^a^	1.62 ± 0.14 ^a^	1.40 ± 0.22 ^a^	1.78 ± 0.28 ^a^	1.50 ± 0.31 ^a^	0–3.6 ^1^
Basophils (%)	0.58 ± 0.45 ^a^	0.20 ± 0.09 ^a^	0.05 ± 0.04 ^a^	0.13 ± 0.05 ^a^	0.03 ± 0.02 ^a^	1.07 ± 0.99 ^a^	0–3 ^1^
RDW (%)	13.84 ± 0.99 ^a^	14.86 ± 2.02 ^a^	13.14 ± 0.21 ^a^	12.80 ± 0.30 ^a^	13.31 ± 0.17 ^a^	15.22 ± 1.33 ^a^	13–18.4 ^3^

RDW: Red cell distribution width. NF—not found. Different letters on the same line indicate statistically significant differences. ^1^ Lima et al., 2014; ^2^ Lapchik, et al., 2009; ^3^ Melo et al., 2012. (Statistical test: ANOVA/Tukey *p* < 0.05).

**Table 4:** Effect of consumption of Natural Fiber-Rich Food Product (NFRFP) on biochemical parameters in rats treated with 1,2-dimethylhydrazine (DMH).

Parameters	Experimental Groups						Reference Value
	Negative Control	Positive Control	NFRFP	Pre-Treatment	Simultaneous	Pos-Treatment	
AST (U/L) ^1^	127.90 ± 8.89 ^b^	108.00 ± 6.22 ^ab^	97.08 ± 3.45 ^a^	110.80 ± 9.10 ^ab^	111.50 ± 5.63 ^ab^	89.45 ± 4.56 ^a^	18–267 ^a^
ALT (U/L) ^1^	45.50 ± 3.52 ^a^	54.48 ± 4.75 ^a^	42.29 ± 2.94 ^a^	57.33 ± 5.15 ^a^	58.29 ± 5.56 ^a^	46.27 ± 4.21 ^a^	34–83 ^a^
Total protein (g/dL) ^1^	6.26 ± 0.15 ^a^	5.91 ± 0.27 ^a^	6.42 ± 0.16 ^a^	6.39 ± 0.10 ^a^	6.25 ± 0.16 ^a^	6.14 ± 0.19 ^a^	5.5–10.4 ^b^
Albumine (g/dL) ^2^	4.19 ± 0.09 ^ab^	3.85 ± 0.23 ^a^	4.36 ± 0.11 ^ab^	4.34 ± 0.05 ^ab^	4.19 ± 0.11 ^ab^	4.11 ± 0.18 ^b^	3.5–4.2 ^a^
Serum urea (mg/dL) ^1^	40.40 ± 1.31 ^a^	38.23 ± 1.39 ^a^	39.15 ± 1.86 ^a^	39.95 ± 1.46 ^a^	39.61 ± 2.46 ^a^	36.56 ± 1.69 ^a^	26–58 ^c^
Creatinine (mg/dL) ^1^	0.42 ± 0.03 ^a^	0.38 ± 0.02 ^a^	0.35 ± 0.02 ^a^	0.43 ± 0.05 ^a^	0.35 ± 0.04 ^a^	0.45 ± 0.03 ^a^	0.24–1.2 ^b^
Uric acid (mg/dL) ^1^	2.04 ± 0.17 ^a^	1.61 ± 0.08 ^a^	1.92 ± 0.17 ^a^	1.83 ± 0.18 ^a^	1.98 ± 0.26 ^a^	1.71 ± 0.13 ^a^	1–3.2 ^b^
Glicose (mg/dL) ^1^	133.0 ± 10.83 ^a^	109.7 ± 8.01 ^a^	124.3 ± 6.96 ^a^	121.6 ± 7.58 ^a^	129.2 ± 10.08 ^a^	119.5 ± 7.27 ^a^	72–193 ^b^
α—amilase (U/L) ^1^	1972 ± 87.57 ^a^	2110 ± 74.16 ^a^	1968 ± 75.71 ^a^	1997 ± 74.98 ^a^	2029 ± 114.00 ^a^	1852 ± 87.03 ^a^	NF
Cholesterol (mg/dL) ^1^	68.95 ± 4.59 ^b^	66.10 ± 3.78 ^ab^	52.28 ± 2.69 ^a^	70.41 ± 1.90 ^b^	62.51 ± 4.97 ^ab^	56.51 ± 4.53 ^ab^	68.9–105.1 ^c^
HDL cholesterol (mg/dL) ^1^	65.93 ± 4.10 ^ab^	57.92 ± 4.92 ^ab^	52.71 ± 2.42 ^a^	68.92 ± 2.28 ^b^	60.25 ± 3.97 ^ab^	51.72 ± 3.22 ^a^	36.6–59.4 ^c^
Triglycerides (mg/dL) ^1^	65.56 ± 7.93 ^a^	96.83 ± 34.67 ^a^	38.88 ± 3.11 ^a^	50.51 ± 5.41 ^a^	52.88 ± 8.44 ^a^	65.25 ± 27.08 ^a^	57.27–106.7 ^c^
Lipase (U/L) ^2^	23.64 ± 15.34 ^a^	29.51 ± 19.36 ^a^	10.26 ± 3.48 ^a^	17.01 ± 10.92 ^a^	2.99 ± 0.22 ^a^	4.21 ± 0.91 ^a^	NF

AST—aspartate aminotransferase; ALT—alanine aminotransferase; NF—not found. Different letters on the same line indicate statistically significant differences. ^a^ Charles River Laboratories, 2008; ^b^ De Lima, 2018; ^c^ Dantas et al., 2006. (Statistical Test: ^1^ ANOVA/Tukey, ^2^ Kruskal-Wallis/Dunn; *p* < 0.05).

## Supplementary Materials

The following are available online at https://www.mdpi.com/article/10.3390/nu13082708/s1, Figure S1: Average food intake (g) of different experimental groups over twelve weeks. NFRFP—Natural Fiber-Rich Food Product. Table S1: Effect of consumption of the Natural Fiber-Rich Food Product (NFRFP) on the weight development of animals submitted to different treatment protocols.
