# Supplementary material for: The Use of Natural Fiber-Rich Food Product Is Safe and Reduces Aberrant Crypt Foci in a Pre-Clinical Model
Source: Nutrients. 2021 Aug 6;13(8):2708. doi: 10.3390/nu13082708 (PMC8401268; doi:10.3390/nu13082708)
Supplement: Supplementary file 1 [file nutrients-13-02708-s001.zip › nutrients-1259046-supplementary.pdf]

Supplementary Materials

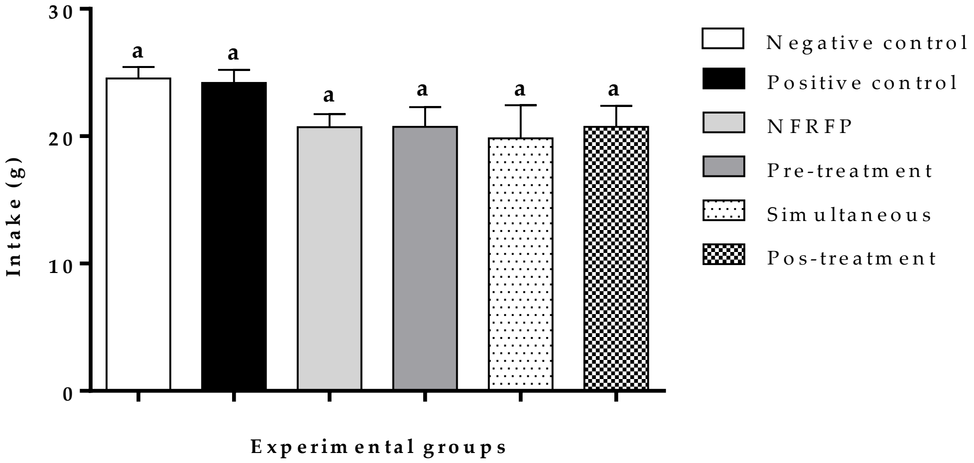

Figure S1. Average food intake (g) of different experimental groups over twelve weeks. NFRFP - Natural Fiber-Rich Food Product. Equal letters on the same line indicate absence statistically significant differences. (Statistical test: ANOVA, $p > 0.05$).

Table S1. Effect of consumption of the Natural Fiber-Rich Food Product (NFRFP) on the weight development of animals submitted to different treatment protocols:

Experimental Groups	Negative Control	Positive Control	NFRFP	Pre-treatment	Simultaneous	Pos-treatment
N	12	11	13	10	9	9
Initial Weight 1	300.6±8.00^{a}	301±9.14^{a}	304.3±7.88^{a}	306.2±8.91^{a}	300.7±8.19^{a}	306.9±9.62^{a}
Final weight[1]	426.8±14.83^{a}	414.5±10.14^{a}	437.8±8.19^{a}	428.6±13.15^{a}	411.9±17.13^{a}	417.6±10.12^{a}
Weight gain[1]	126.2±19.09^{a}	113.5±15.47^{a}	133.5±11.88^{a}	119.0±16.81^{a}	111.2±15.86^{a}	110.7±13.68^{a}
Absolute weight						
Heart[1]	1.14±0.05^{a}	1.14±0.03^{a}	1.15±0.03^{a}	1.14±0.05^{a}	1.19±0.05^{a}	1.17±0.03^{a}
Lungs[1]	1.70±0.07^{ab}	1.71±0.05^{ab}	1.72±0.05^{ab}	1.64±0.07^{a}	1.62±0.05^{a}	1.93±0.07^{b}
Liver[1]	13.20±0.47^{a}	14.29±0.82^{a}	14.17±0.35^{a}	13.81±0.50^{a}	13.92±0.87^{a}	13.64±0.67^{a}
Spleen[2]	0.75±0.06^{a}	0.98±0.26^{a}	0.77±0.03^{a}	0.74±0.04^{a}	0.83±0.10^{a}	0.96±0.25^{a}
Kidneys[2]	2.88±0.12^{a}	2.94±0.08^{a}	3.05±0.08^{a}	2.93±0.12^{a}	2.85±0.17^{a}	3.32±0.66^{a}
Right epididymis[1]	0.42±0.02^{a}	0.53±0.13^{a}	0.50±0.04^{a}	0.48±0.05^{a}	0.58±0.10^{a}	0.49±0.04^{a}
Right testicle[1]	1.72±0.07^{a}	1.53±0.12^{a}	1.74±0.03^{a}	1.68±0.06^{a}	1.51±0.16^{a}	1.59±0.05^{a}
Relative weight						
Liver[1]	3.10±0.05^{a}	3.51±0.31^{a}	3.24±0.05^{a}	3.25±0.06^{a}	3.38±0.14^{a}	3.27±0.15^{a}
Heart[1]	0.27±0.01^{a}	0.28±0.01^{a}	0.26±0.01^{a}	0.27±0.00^{a}	0.29±0.01^{a}	0.28±0.01^{a}
Lungs[1]	0.40±0.02^{ab}	0.42±0.02^{ab}	0.39±0.01^{a}	0.38±0.01^{a}	0.39±0.01^{a}	0.46±0.01^{b}
Kidneys[2]	0.67±0.01^{a}	0.71±0.02^{a}	0.70±0.01^{a}	0.69±0.02^{a}	0.69±0.02^{a}	0.79±0.15^{a}

Spleen[2]	0.18±0.01[a]	0.25±0.08[a]	0.18±0.01[a]	0.17±0.01[a]	0.20±0.03[a]	0.23±0.06[a]
Right epididymis[1]	0.43±0.02[a]	0.53±0.13[a]	0.50±0.04[a]	0.48±0.05[a]	0.58±0.10[a]	0.49±0.04[a]
Right testicle[1]	1.85±0.38[a]	1.785±0.49[a]	1.43±0.12[a]	1.69±0.24[a]	1.57±0.31[a]	1.66±0.22[a]

NFRFP - Natural Food Product Rich in Fiber. Different letters on the same line indicate statistically significant differences (Statistical Test: 1ANOVA / Tukey, 2Kruskal-Wallis / Dunn, p> 0.05).
